# An unusual bacterium causing a brain abscess.

**DOI:** 10.3201/eid0701.010127

**Published:** 2001

**Authors:** D N Atapattu, D P Jayawickrama, V Thevanesam


Michael Dan,* Francesca Poch,* Daniel Shpitz,† and Bracha Sheinberg†
*Infectious Disease Unit, E. Wolfson Hospital, Holon, Israel, and †Maccabi Health Services, Rishon-le-Zion, Israel

### References

1. The Israel Center for Diseases Control. Notifiable infectious disease in Israel, 1951-1995. Israel: State of Israel: Ministry of Health; 1996. pub. no. 201.
2. District Health Office, Tel-Aviv. Report on emerging issues, July-December 1999. State of Israel: Ministry of Health; Jan 10, 2000.
3. Centers for Disease Control and Prevention. Guidelines for treatment of sexually transmitted diseases. Mor Mortal Wkly Rep 1998;47(RR-1):1-116.
4. Tapsall JW, Shultz TR, Phillips EA. Characteristics of *Neisseria gonorrhoeae* isolated in Australia showing decreased sensitivity to quinolone antibiotics. Pathology 1992;24:27-31.
5. Quinn TC. The impact of antimicrobial resistance on the treatment of sexually transmitted diseases. Infect Dis Clin North Am 1997;11:884-904.
6. Knapp JS, Fox KK, Trees DL, Whittington WL. Fluoroquinolone resistance in *Neisseria gonorrhoeae*. Emerg Infect Dis 1997;3:33-8.
7. Tanaka M, Nakayama H, Haraoka M, Saika T, Kobayashi I, Naito S. Antimicrobial resistance of *Neisseria gonorrhoeae* and high prevalence of ciprofloxacin-resistant isolates in Japan, 1993 to 1998. J Clin Microbiol 2000;38:521-5.
8. Tapsall JW, Limnios EA, Thacker C, Donovan B, Lynch SD, Kirky LJ, et al. High-level quinolone resistance in *Neisseria gonorrhoeae*: a report of two cases. Sex Transm Dis 1995;22:310-11.
9. Centers for Disease Control and Prevention. Fluoroquinolone-resistant *Neisseria gonorrhoeae*–San Diego, California, 1997. Mor Mortal Wkly Rep 1998;47:405-8.
10. Dillon B, Hecht FM, Swanson M, Goupil-Sormany I, Grant RH, Chesney MA, et al. Primary HIV infections associated with oral transmission. Presented at the 7th Conference on Retroviruses and Opportunistic Infections, San Francisco, Jan 30–Feb 2, 2000 (Abstract 473).

### An Unusual Bacterium Causing a Brain Abscess

**To the Editor:** Intracranial abscesses are an important cause of illness and death in a neurologic/neurosurgical unit. Early presumptive clinical diagnosis supported by radiologic evidence (computerized axial tomography [CAT] scan and magnetic resonance imaging) is the mainstay of diagnosis (1). Abscess contents are aspirated under stereotaxic guidance and cultured to isolate causative organisms and determine their antibiotic sensitivities. Organisms isolated from brain abscesses are usually streptococci, anaerobic and facultative gram-negative bacilli, staphylococci, or pseudomonads (2).

A 24-year-old male farmer came to us with progressive headache, dizziness, and a low-grade fever of 2 weeks' duration. He had had a pimple on his right cheek approximately 3 weeks before, which had discharged "bluish" pus on forcible evacuation and subsequently healed without treatment. No focal neurologic signs were detected on physical examination. Because an intracranial space-occupying lesion was suspected, a lumbar puncture was withheld. Later, a CAT scan of the patient's head revealed a right-sided temporoparietal space-occupying lesion approximately 3 cm in diameter, suggestive of a unilocular brain abscess. The abscess was needle aspirated under stereotaxic guidance, and the pus was cultured aerobically and anaerobically. After 24 hours of aerobic incubation on MacConkey agar at 37°C, a pure growth of violet-colored colonies appeared, identified as *Chromobacterium violaceum* by the 20E API system (Biomerieux, France).

Other initial laboratory findings were as follows: blood leukocyte count, 16,200 cells/µL (84% neutrophils, 15% lymphocytes, 1% eosinophils); erythrocyte sedimentation rate (Westergren method), 22 mm/hour; C-reactive protein concentration, 96 mg/L; and fasting blood sugar concentration, 5.1 mmol/L. Blood urea and C-reactive protein concentrations after 3 weeks of antibiotic treatment were 4.6 mmol/L and <6 mg/L, respectively.

The organism was sensitive to imipenem and ciprofloxacin and resistant to cefotaxime and ceftriaxone, by the Stokes comparative disk-diffusion antibiotic sensitivity testing method (3). Ciprofloxacin (as lactate) was administered intravenously, 400 mg twice a day, for 4 weeks. Repeated CAT scans, clinical symptoms, and serial C-reactive protein levels indicated rapid regression of the abscess followed by complete cure.

*C. violaceum* is a gram-negative bacillus present in soil and aquatic environments of tropical and subtropical countries or regions such as Trinidad, Guyana, India, Malaysia, Florida, and South Carolina. It is a bacterium of low virulence, occasionally causing skin infections and disseminated disease involving multiple organs in immunocompromised patients. In such cases the disease can mimic septicemic melioidosis (4,5).

In this previously healthy patient, infection probably originated from the facial abscess. The patient was negative for HIV antibody (Serodia), had no history of diabetes mellitus or other compromising illnesses, and had no evidence of immunodeficiency. In a previous case of disseminated *C. violaceum* infection in a young patient, postmortem findings revealed numerous cortical infarcts and hemorrhages (6). Our isolate from a brain abscess is yet another case of a relatively avirulent saprophytic microorganism resulting in a deep-seated infection in a well-nourished, previously healthy person.

**Dhammika Nanda Atapattu,***
**Dhammika Priyal Jayawickrama,*† and**
**Vasanthi Thevanesam***
*University of Peradeniya, Peradeniya, Sri Lanka;
†General Hospital, Kandy, Sri Lanka

### References

1. Mathisen GE, Johnson JP. Brain abscess. Clin Infect Dis 1997;25:763-81.
2. Mandell GL, Bennett J, Dolin R. Principles and practice of infectious diseases. 4th ed. New York: Churchill Livingstone; 1995: p. 887-99.
3. Stokes EJ, Ridgway GL, Wren MWD. Clinical microbiology. 7th ed. London: Edward Arnold; 1993: p. 239-50.
4. Murray PR, Baron EJ, Pfaller MA, Tenover FC. Manual of clinical microbiology. 6th ed. Washington: ASM press; 1995: p. 503.
5. Mitchell RG. In: Parker MT, Duerden BI, editors. Miscellaneous bacteria. Topley and Wilson's principles of bacteriology, virology and immunity, Vol. 2. 8th ed. London: Edward Arnold; 1990: p. 589-91.
6. Ti TY, Tan WC, Chong APY. Non fatal and fatal infections caused by *Chromobacterium violaceum*. Clin Infect Dis 1993;17:505-7.

### First Glycopeptide-Resistant *Enterococcus faecium* Isolate from Blood Culture in Ankara, Turkey

**To the Editor:** Glycopeptide-resistant enterococci infections are a major problem in hospitals. Infection or colonization by vancomycin-resistant enterococci was first reported in France (1) and the United Kingdom (2); since then, these organisms have been reported throughout the world. In Turkey, vancomycin and teicoplanin have been used to treat serious methicillin-resistant *Staphylococcus aureus* and ampicillin-resistant enterococci infections.

We describe the case of an acute myelocytic leukemia patient with vancomycin-resistant enterococci bloodstream infection. This is the first glycopeptide-resistant *Enterococcus faecium* isolate from our hospital and from Ankara, Turkey. The patient had not been cared for at another institution.

A 68-year-old man, hospitalized with acute myelocytic leukemia, had fever episodes during the neutropenia following three courses of remission-induction chemotherapy (daunorubicin+cytosine arabinoside). A combination of antibiotics including vancomycin, ceftazidime (sometimes imipenem), and amikacin was administered with different regimens during the 5 months of hospitalization. Blood, urine, and rectal swab cultures during this period were positive for different *Enterobacteriaceae* spp. but always negative for vancomycin-resistant enterococci. For long-term hospitalizations, our center routinely performs surveillance rectal swab cultures. At the end of month 5, *E. faecium* was isolated from the blood cultures, just 1 day before the patient's death.

The strain was identified by conventional methods, commercial automatic systems (API Strep-Biomerièux, France), and polymerase chain reaction. Susceptibility patterns showed that the isolate was resistant to all antibiotics except ciprofloxacin and levofloxacin. When the E-test was used, MIC levels for vancomycin, teicoplanin, ciprofloxacin, and levofloxacin were 256 µg/mL, 64 µg/mL, 0.75 µg/mL, and 1.5 µg/mL, respectively. *VAN-A1* and *Van-A2* type resistance genes were detected by polymerase chain reaction. Hacettepe University microbiology laboratories confirmed these results (3,4).

After this strain was isolated, 1,266 stool and 176 rectal swab samples were taken from hospital personnel in three sessions $\geq$1 week apart, and patients were tested for vancomycin-resistant enterococci. Swab cultures from all environmental surfaces (bed rails, bedside commodes, carts, charts, doorknobs, faucet handles) were also examined. We injected all samples with 5% sheep blood agar with vancomycin (6 mg/L); vancomycin-resistant *E. faecium* was not identified in any sample.

